# Expectations of pain and functioning in patients with musculoskeletal disorders: a cross-sectional study

**DOI:** 10.1186/s12891-016-1386-z

**Published:** 2017-01-26

**Authors:** Sigrid Skatteboe, Cecilie Røe, Morten Wang Fagerland, Lars-Petter Granan

**Affiliations:** 1 Deparment of Physical Medicine and Rehabilitation, Oslo University Hospital, Oslo, Norway; 2 Faculty of Medicine, University of Oslo, Oslo, Norway; 3 Oslo Centre for Biostatistics and Epidemiology, Research Support Services, Oslo University Hospital, Oslo, Norway; 4 Department of Pain Management and Research, Division of Emergencies and Critical Care, Oslo University Hospital, Oslo, Norway; 5 Ullveien 19b, 0791 Oslo, Norway

**Keywords:** Back pain, Neck pain, Shoulder pain, Musculoskeletal pain, Treatment outcome

## Abstract

**Background:**

Research has suggested that patient expectations are associated with treatment outcome and evolve along with patient communication within the musculoskeletal field. However, few studies have investigated if or how physical medicine and rehabilitation (PMR) consultations affect the attending patients’ expectations regarding pain and functional improvement. Hence, the aims of the present study were to compare patient expectations regarding pain and functional improvement before and after a PMR consultation and to assess patient characteristics, including diagnosis, that could perhaps predict changes in expectations.

**Methods:**

The study design was cross-sectional. Eligible participants were first-time patients with neck/back or shoulder complaints who were referred to a PMR outpatient clinic between January and June 2013. Questionnaires (the Patient Shoulder Outcome Expectancies, or PSOE, questionnaire and a numeric rating scale, or NRS) focused on expectations regarding pain and functioning were completed immediately prior to and after a consultation with a PMR specialist.

**Results:**

In total, 257 patients were included. In total, 24% of the subjects expected a more positive outcome after the PMR consultation compared with before the consultation, while 10% of the subjects exhibited a negative change in expectations. Few patient characteristics other than sick leave were associated with changes in expectations; however, patients with shoulder complaints seemed to be more optimistic than patients with neck/back complaints.

**Conclusion:**

Expectations can be influenced by a single specialist consultation. Among clinical prognostic factors, only sick leave influenced the change expectations. However, patients with shoulder complaints seemed to be more optimistic than patients with neck/back complaints.

**Trial registration:**

The study was approved by the Data Protection Office at Oslo University Hospital, 2012/2574. ISRCTN registration: 40963362 (registered retrospectively 12.12.2016)

**Electronic supplementary material:**

The online version of this article (doi:10.1186/s12891-016-1386-z) contains supplementary material, which is available to authorized users.

## Background

An expectation can be defined as “a person’s subjective opinion about an outcome” [1]. From a medical perspective, many symptoms and diagnoses are often accompanied by expectations about the medical complaint, the subsequent treatment [2], and the prognosis and outcome [3]. Expectations are typically individual and heterogeneous. However, conceptualized categories such as socioeconomic background [4], previous health experiences [5], personality and emotional distress [6] and musculoskeletal pain [7] can affect expectations.

Patient expectations are notable for several reasons, but predominantly due to the suggested association with treatment outcomes [8]. This relationship is mainly observed within the musculoskeletal field in patients with low back pain [9], neck pain [10] and shoulder pain [8]. In a systematic review by Mondloch et al., positive treatment expectations were associated with improved health outcomes in 15 of 16 studies [11]. Unrealistic expectations, whether high or low, are suggested to negatively influence outcomes [12]. This concept has inspired hypotheses regarding clinical utilizations of expectations, e.g., as described by Mondloch et al. [11] and Myers et al. [13], suggesting that adjustments of negative, unrealistic and/or non-beneficial expectations [14] could improve outcomes [9]. However, few clinical trials have examined these hypotheses. Mancuso and coworkers [15] attempted to modify expectations in a randomized controlled trial (RCT), and their results suggested that expectations are adjustable. This trial was, however, a surgical trial, so the results may be less applicable to conservative approaches [16]. Additionally, in a systematic review of patient-physician relationships, ten of the 19 included studies demonstrated that positively enhancing patient expectations significantly improved health outcomes [17]. However, this review did not specifically target musculoskeletal patients or expectations specifically regarding pain and functioning.

Furthermore, patient expectations have been noted to be relevant in patient communication, especially in reducing misunderstanding [18], increasing satisfaction [19] and encouraging shared decision making [20]. Patients with musculoskeletal conditions with pain and functional complaints have been shown to require careful provision of information [21], and the inclusion of a discussion of expectations in clinical consultations could be useful for further improvement of patient communication and care. Health care professionals have been shown to have a strong influence on patient attitudes and beliefs [22], and it is likely that the dialog during a consultation can influence patient expectations. Finally, little is known about how expectations vary among different patient groups, and we were unable to find literature comparing different joint conditions within the musculoskeletal system in this context.

The aims of the present study were to compare expectations regarding pain and function before and after a consultation with a physical medicine and rehabilitation (PMR) physician and to assess whether changes in expectations varied among patients with neck/back or shoulder complaints, and/or were associated with patient characteristics.

## Methods

### This study had a cross-sectional design

#### Patients

The Norwegian health care system is run by the National Insurance Scheme (Folketrygden, or NIS), which covers all residents. Hence, patients are only charged portions of the total cost, as regulated by the government. There is also an upper limit to how much each patient can be charged every year, and the government covers costs beyond that limit. All citizens are assigned to a general practitioner (GP), which can be changed twice per year if the patient wishes. The GPs are responsible for referring patients to specialized care, often located in public hospitals. Oslo University Hospital (OUS) not only covers the capital area but also obtains regional assignments, resulting in coverage of 0.5 and 2.6 million inhabitants, respectively. The OUS PMR clinic consists of both an outpatient clinic and a hospital ward. The outpatient clinic receives patients with musculoskeletal complaints associated with pain/functional problems; clear surgical referrals are not accepted. Only patients > 15 years of age are permitted. Approximately 20 patients with neck/back and shoulder complaints visit the outpatient clinic daily.

The present study was based on information extracted from questionnaires completed by patients with neck/back and shoulder complaints at the PMR outpatient clinic. A sample of patients referred to the PMR outpatient clinic between January and June 2013 (18 weeks) were considered eligible for inclusion. The exclusion criteria were previous visits to the clinic, a lack of a neck/back/shoulder diagnosis and a lack of consent. An interpreter was utilized to help non-Norwegian-speaking patients to understand and complete the forms if the interpreter was pre-booked for the consultation. The first author was responsible for both including patients and administrating the forms regarding expectations. Not all potential eligible patients were included due to the logistic limitations of having only one administrative person involved.

#### Procedures

As part of the clinical routine, all primary attending patients at the PMR outpatient clinic received a general questionnaire by mail, together with the appointment letter. This questionnaire recorded general information (described below) and was filled prior to the consultation day. Patients eligible for the current study obtained a written letter providing information about the study and a consent letter on the day of the consultation. Consenting participants thereafter received two forms regarding expectations. One was completed immediately before the consultation, and the second was completed directly after the consultation (approximately 1 h later). These two forms considered the present status of pain and function and expectations regarding improvement in pain and function (described below and found in Additional file 1). The PMR physicians did not receive any instructions regarding the study.

#### Assessments

The primary outcome, namely, expectations, was measured using the Norwegian version of the Patient Shoulder Outcome Expectancies (PSOE) [8] questionnaire. This measure contains questions about expectations regarding the overall problem, the specific pain, and one’s ability to move one’s shoulder/neck/back during the next month. The three questions are scored on a six-point numeric rating scale (NRS), ranging from one (“much worse”) to six (“much better”) [8]. Evidence for the unidimensionality of this measure was obtained from a confirmatory factor analysis in which one factor accounted for 89% of the item variance, and the internal consistency was calculated, with a Cronbach’s alpha of 0.94 [8]. The modification of the original English-language, shoulder-problem related version of the questionnaire for use in Norwegian and in a neck/back problem population has previously been documented [23]. Expectations (PSOE) were measured both before and after the consultation.

Three 11-point NRSs (11NRS) were included to define the present status before the consultation. The scales recorded pain during rest, pain during activity, and physical functioning. The scales were scored between zero, indicating “no pain/no movement limitations,” and ten, indicating “worst possible pain/no movement possible.”

The general questionnaire sent to the patients prior to the consultation and returned on the day of the consultation included the following:

The Hopkins Symptom Checklist-10 (HSCL-10) [24] was included as a measurement of emotional distress. The HSCL-10 exhibits reliable sensitivity and specificity and has been tested in the Norwegian population [25]. A Cronbach’s alpha of 0.88 has been demonstrated [25], which is considered a reliable score [26]. Each question has four response categories (“not at all,” “a little,” “quite a bit,” and “extremely”) and is scored from one to four. Missing values were replaced with the mean value of the other items if three or fewer items were missing. A mean score was not calculated for subjects with more than three missing values (n = 17). A mean value higher than the suggested cut-off score of 1.85 suggested elevated emotional distress [25].

The pain distribution for the previous 14 days was indicated schematically on a drawing of the body. The number of pain sites (NPS) on the drawing was recorded according to the protocol of Kamaleri et al. [27].

Demographic data included age and sex, language (Norwegian/other), marital status (married/partner/single), level of education (>13 years), sick leave due to neck/back/shoulder problems (yes/no), smoking (yes/no) and daily use of analgesics (yes/no). The number of days (“time of waiting”) between referral and the PMR consultation was also recorded, as was the International Classification of Diseases, Tenth Edition (ICD-10) diagnosis selected by the physician (specific or not, with the classification provided in Additional file 2).

#### Statistics

Patient characteristics were compared between those completing all forms and the non-completers using independent-sample t-tests.

Expectations were scored according to the protocol of O’Malley et al. [8]: the scores on the three PSOE questions were summed, yielding a maximum total score of 18. A score of nine equaled an expectation of no change in status. Scores below nine indicated an expectation of aggravation of status, while scores above nine indicated an expectation of improvement. Changes in expectations were defined as the difference in PSOE values before and after the PMR consultation. This difference was classified as “unchanged” if expectations were identical before and after the consultation (difference score of zero). “Improved” implied that expectations regarding pain and functional status were more optimistic after the consultation compared with before (positive difference score). In contrast, “worse” indicated that expectations were more pessimistic after the consultation (negative difference score). A test for marginal homogeneity [28] was used to compare the distributions of the “improved,” “unchanged” and “worse” classifications before and after the consultation. The null hypothesis was that the proportions of patients reporting “improved,” “unchanged,” and “worse” expectations were the same before and after the PMR consultation (marginal homogeneity). A *p*-value < 0.05 for the test of marginal homogeneity indicated a qualitative change in expectations, such as from “unchanged” to “improved.” A mean value was calculated from the three 11NRS, from here on defined as “PainFunction.”

Univariable and multivariable linear regression models were used to assess the relationship between the change in expectations (PSOE) from before to after the PMR consultation (dependent variable) and the independent variables, which included language, marital status, level of education, sick leave due to this problem, smoking, daily use of analgesics, the number of days between referral and the PMR consultation, and the ICD-10 diagnosis (variable categorization specified above). A joint-specific variable (shoulder versus neck/back problem) was also included as an independent variable. All variables were checked for deviations from normality, non-linear effects, multicollinearity and homoscedasticity. Firstly, univariable analyses were performed on each of the independent variables. Secondly, all independent variables were included in a multivariable model.

In addition to the regression analyses described above, the difference in expectations among patients with shoulder complaints compared with patients with neck/back complains was assessed using independent-sample t-tests.

The sample size was based on estimation for medium-effect regression models, as described by Green [29], with N > 104 + k (number of independent variables) = a minimum of 118 included patients, but additional participants were included for supplementary prediction analyses (shoulder versus neck/back complaints comparison) and to account for drop out during the planned 6-month follow-up. At least 15 subjects were included per independent variable in all regression models.

The level of statistical significance was set to 0.05 for both t-tests and regression models. All statistical analyses were performed using SPSS version 22.0 (IBM Corp. Statistics for Macintosh, Armonk, NY, USA).

## Results

**Fig. 1:**
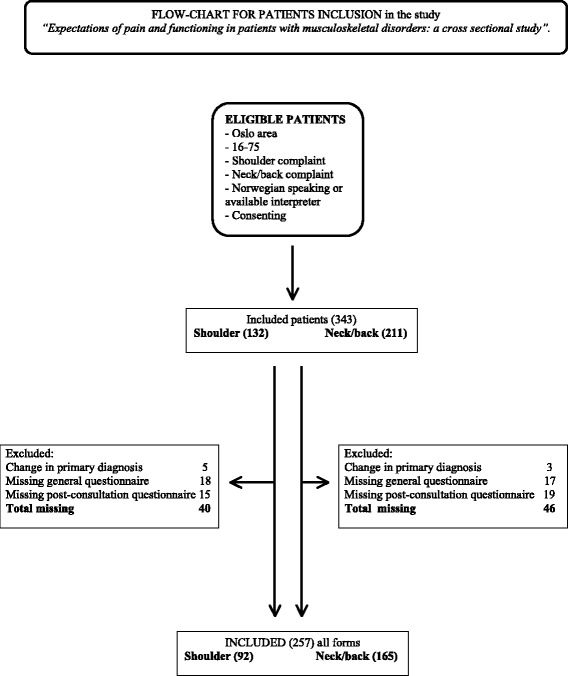
FLOW-CHART FOR PATIENTS INCLUSION in the study. “Expectations of pain and functioning in patients with musculoskeletal disorders: a cross sectional study”

Five patients did not consent to participate and thus were not included in the study. In total, 343 patients were eligible for inclusion. Only 257 patients completed all questionnaires (the general questionnaire and the questionnaires both before and after the consultation). Hence, the analyses were performed on 257 patients, with 165 with neck/back complaints and 92 with shoulder complaints. An overview of the inclusion process can be found in the flow chart (Fig. 1). The non-included participants (patients with incomplete questionnaires (n = 78) and patients with a changed primary diagnosis (n = 8)) did not differ significantly from the included patients, except in terms of the time of waiting. The remaining comparisons between completers and non-completers are found in Table 1. However, most of the non-completers did not fill out the general questionnaire, and hence, we did not have complete patient characteristics for this group. The mean age of the included patients was 49.4 years (SD 14.9), and 51.4% of the patients were females. Patients with neck/back complaints differed from patients with shoulder complaints on four variables: greater use of analgesics; less education; a greater NPS; and a higher HSCL-10 score (1.95), with a mean value above the cut-off value, in contrast to the patients with shoulder complaints (1.71). Finally, the patients with neck/back complaints seemed to be less optimistic after the consultation compared with the patients with shoulder complaints. All patient characteristics are presented in Table 1.

**Table 1:** Characteristics of patients with (257) and without (86) complete questionnaires

(n) % / Mean (SD)	Neck (*n* = 58)/back (*n* = 107)	Shoulder (*n* = 92)	p-value (1)	Total (*n* = 257)	Non-completing	p-value (2)
	(165) 64.2%	(92) 35.8%		257	86	
Sex (female)	(84) 50.9%	(48) 52.2%	0.949	(132) 51.4%	(44) 52.3%	0.462
Age	50.1 (15.6)	48.4 (13.2)	0.287	49.4 (14.9)	46.6 (12.9)	0.875
Smokers (daily)	(29) 18.0%	(20) 21.5%	0.609	(47) 19.0%	(12) 18.8%	0.971
Education (higher)	(47) 51.7%	(47) 50.6%	0.014**	(110) 43.8%	(39) 51.3%	0.298
Use of analgesics (daily)	(69) 44.2%	(19) 20.7%	0.009**	(88) 35.8%	(15) 21.4%	0.148
Sick leave (yes/no)	(72) 49.3%	(39) 44.8%	0.509	(111) 47.6%	(41) 48.2%	0.063
Language (Norwegian)	(132) 85.7%	(78) 86.7%	0.837	(210) 86.1%	(52) 69.3%	0.863
Marital status (married/partner/single)	(62) 67.4%	(63) 67.7%	0.486	(173) 70.0%	(53) 62.4%	0.452
Time of waiting (days)	69.6 (40.4)	65.8 (35.9)	0.454	68.2 (38.8)	82.3 (47.1)	0.014**
HSCL-10 > 1.85	(68) 50.0%	(33) 36.7%	0.003**	(101) 42.8%	(18) 41.9%	0.991
PSOE exp. before consultation	8.7 (3.3)	8.4 (3.2)	0.417	8.6 (3.2)	8.9 (3.3)	0.533
PSOE exp. after consultation	8.2 (2.7)	7.1 (2.4)	0.003**	7.7 (2.6)	7.9 (2.4)	0.788
Pain and function*^1^	5.4 (2.1)	5.1 (2.0)	0.300	5.3 (2.1)	5.1 (2.2)	0.586
Number of pain sites	4.0 (2.2)	3.0 (1.8)	0.000**	3.6 (2.1)	3.52 (3.4)	0.762
Back-specific diagnosis*^2^	(64) 59.8%					
Neck-specific diagnosis*^2^	(26) 44.8%					
Subacromial conditions*^2^		(26) 28.3%				
Adhesive capsulitis*^2^		(14) 15.2%				
Degeneration/arthrosis*^2^		(2) 2.2%				
Other shoulder conditions*^2^		(50) 54.3%				

(1) Comparing of the neck/back and shoulder population

(2) For comparing patients with and without complete questionnaires

*HSCL* Hopkins Symptom Checklist-10 (HSCL-10), cut-off of 1.85, *PSOE* Patient Shoulder Outcome Expectancies, *Exp.* expectations

**Significant at *p* < 0.05

*^1^PainFunction: mean 11NRS pain and functioning baseline scores

*^2^Diagnosis categorization in additional file 2

### Changes in expectations

In total, 24% of patients had more positive expectations after the consultation, and 9% of patients had more negative expectations after the consultation, as measured by the PSOE (Table 2). A more positive shift was observed in the patients with shoulder complaints than in the patients with neck/back complaints.

**Table 2:** Patient expectations regarding pain and functional status (PSOE) before (rows) and after (columns) the PMR consultation

All patients				
		Exp.^*1^ after cons.^*2^		
Exp. ^*1^ before cons. ^*2^	n (%)	Better	Unchanged	Worse
	Better	90 (35.0)	20 (7.8) ^(w*3)^	1 (0.4) ^(w*3)^
	Unchanged	31 (12.1) ^(b*4)^	57 (22.2)	3 (1.2) ^(w*3)^
	Worse	18 (7.0) ^(b*4)^	12 (4.7) ^(b*4)^	25 (9.7)
	No change: 66.9%, better: 23.9%, worse: 9.4%			
	*p* < 0.0001 (test for marginal homogeneity)			
Patients with neck/back complaints				
		Exp.^*1^ after cons.^*2^		
Exp. ^*1^ before cons. ^*2^	n (%)	Better	Unchanged	Worse
	Better	52 (31.5)	14 (8.5) ^(w*3)^	1 (0.6) ^(w*3)^
	Unchanged	17 (10.3) ^(b*4)^	42 (25.5)	3 (1.8) ^(w*3)^
	Worse	9 (5.5) ^(b*4)^	8 (4.8) ^(b*4)^	19 (11.5)
	No change: 69.1%, better: 20.6%, worse: 10.9%			
	*p* < 0.0013 (test for marginal homogeneity)			
Patients with shoulder complaints				
		Exp.^*1^ after cons.^*2^		
Exp. ^*1^ before cons. ^*2^	n (%)	Better	Unchanged	Worse
	Better	38 (41.3)	6 (6.5) ^(w*3)^	0 ^(w*3)^
	Unchanged	14 (15.2) ^(b*4)^	15 (16.3)	0 ^(w*3)^
	Worse	9 (9.8) ^(b*4)^	4 (4.3) ^(b*4)^	6 (6.5)
	No change: 64.1%, better: 29.3%, worse: 6.5%			
	*p* < 0.0004 (test for marginal homogeneity)			

^*1^ *Exp.* expectations, ^*2^ *cons.* consultation, ^*3^ *w* worse, *b* ^*4^ better

### Predictors of changes in expectations

Few patient characteristics predicted changes in expectations from before to after the consultation (Table 3). Only having a neck/back-related complaint compared with a shoulder-related complaint as well as sick leave and baseline expectations were statistically significant as predictors. The combined included variables described 44.2% of the total variance (R^2^) in changes in expectations.

**Table 3:** Univariable and multivariable linear regression models for predictors of positive change in expectations (PSOE; dependent variable) after the consultation

	Univariable models			Multivariable model		
Predictor (independent variable)	Estimated regression coefficient	95% CI	p-value	Estimated regression coefficient	95% CI	p-value
Number of pain sites	0.021	−0.138 to 1.180	0.796	0.000	−0.148 to 0.147	0.996
Marital status (married/partner/single)	−0.359	−1.105 to 0.386	0.343	−0.007	−0.644 to 0.631	0.983
Pain and function*^1^	0.171	0.009 to 0.333	0.039**	0.013	−0.159 to 0.185	0.881
Smokers (daily)	0.105	−0.766 to 0.977	0.812	0.193	−0.581 to 0.968	0.624
Use of analgesics (daily)	0.024	−0.264 to 0.312	0.870	−0.095	−0.387 to 0.196	0.520
Sex (female)	−0.594	−1.261 to 0.073	0.081	−0.295	−0.903 to 0.312	0.339
Language (Norwegian)	0.330	−0.664 to 1.323	0.514	−0.436	−1.326 to 0.453	0.335
HSCL-10 > 1.85	0.471	−0.093 to 1.036	0.101	−0.387	−0.996 to 0.221	0.200
Time of waiting (days)	0.005	−0.004 to 0.014	0.248	0.005	−0.003 to 0.012	0.200
Education (higher)	0.200	−0.144 to 0.544	0.254	0.206	−0.105 to 0.517	0.192
Age	0.017	−0.005 to 0.040	0.127	0.019	−0.002 to 0.039	0.077
Sick leave (yes/no)	0.389	−0.315 to 1.093	0.277	0.738	0.080 to 1.395	0.028**
Neck/back or shoulder diagnosis*^2^	0.662	−0.033 to 1.357	0.062	0.733	0.105 to 1.361	0.022**
PSOE exp. Before consultation	0.520	0.438 to 0.602	0.000**	0.548	0.453 to 0.644	0.000**

*HSCL* Hopkins Symptom Checklist-10 (HSCL-10), cut-off of 1.85, *PSOE* Patient Shoulder Outcome Expectancies, *Exp.* expectations

**Significant at *p* < 0.05

*^1^PainFunction: mean 11NRS pain and functioning baseline scores

*^2^Diagnosis categorization in additional file 2

## Discussion

One quarter of the patients changed their expectations in a positive direction after the consultation. This optimistic change was unexpected, considering the chronicity surrounding patients referred to specialist PMR clinics. Expectations should, in our opinion, be considered malleable. We were unable to find comparable literature regarding this alteration in patient expectations. However, if only pre-consultation expectations are considered, a study by Boonstra et al. [30] suggested that 61% of rehabilitation patients expected less pain and that 53% expected more activity prior to intervention, which are similar rates to those presented in our study.

In contrast, 10% of the patients in our study were more pessimistic regarding future pain and functional status after the first consultation. This negative alteration may have been due to clarification of an unrealistic prior expectation. Attending patients may have inappropriate insights into their own condition and present unrealistic assumptions for the upcoming process and prognosis. In 2008, Lurie et al. [2], for instance, found that the majority of patients with lumbar disk herniation preferred and expected surgery. It is likely that not only patients with lumbar disk pathology but also a variety of patients with neck/back or shoulder complaints do not expect or prefer a non-surgical approach. All unmet presumptions regarding treatment preferences, radiology, and physiotherapy/training interventions and other disagreements related to follow-up care may influence patient expectations. Furthermore, many patients are inadequately informed about their condition, regardless of the duration of their condition. The first PMR consultation may uncover unrealistic expectations based on imprecise insight. Unrealistic high or low expectations have been demonstrated to negatively influence treatment outcomes [12]. Despite this negative shift in 10% of the patients in the current study, this re-alignment of expectations could be beneficial for the upcoming care and process. Personalized information has previously been demonstrated to be vital for the understanding and handling of back pain conditions [31]. Dissimilarities between the expectations of the patient and the physician can be a disadvantage [32], which emphasizes that patient communication, including regarding expectations, should be prioritized.

Two thirds of the patients in the present study had unchanged expectations. This result is not surprising, considering the chronicity surrounding these complaints. Sanderson et al. [33] investigated how expectations changed over 3 months in subjects with low back pain, and no change was found. This finding suggests that expectations may be fairly stable, at least over shorter time spans. A prolonged duration may reduce expectations regarding recovery, and pain and movement limitations may challenge positive attitudes. However, the aforementioned study is not directly comparable to our study due to the specific interventions and method of randomization of patients used. Perhaps the lack of change within both studies implies that expectations are fairly stable in patients with chronic conditions. Vasseljen et al. [34] reported that the majority of back pain patients experienced little change in their pain status over 1 year.

Nevertheless, an intriguing question is what a “proper” change in expectation would be. The literature refers to realistic expectations [12]. Two persons with similar conditions could therefore be dissimilar in their change in expectations compared to the initial expectations. It is challenging to predict what a realistic expectation is, few musculoskeletal diagnoses have a pre-determined progression, and individual variation must also be expected.

In the current study, the regression analyses revealed that few clinical or demographic factors were associated with changes in expectations regarding improvement in pain and function: only sick leave and diagnosis were found to be statistically significant (as discussed below). Patients on sick leave due to the neck/back/shoulder problem had increased positive expectations after the consultation. Unfortunately, directly comparable literature considering changes in expectations is scarce, and we were unable to find comparable literature regarding sick leave. However, Reme et al. [35], among others, indicated that pessimistic expectations delayed work return in a low back pain population. Perhaps this subgroup of patients should receive specific communication addressing their expectations. However, the homogeneity of the group, with few significantly divergent predictors, is surprising because the literature frequently suggests associations between expectations and patient characteristics and/or socioeconomic factors, as summarized by Bialosky et al. [16], among others. However, our study investigated changes in expectations, and the lack of similar literature challenges interpretation. Perhaps the absence of divergent predictors is caused by unrevealed factors affecting the change in expectations that were not considered here. Topics of possible interest include aspects of personality, other psychological traits, self-efficacy and patients’ explanatory models. It has also been previously demonstrated that social relationships and previous experiences with health care are important [36], neither of which was examined here.

A final aspect is the comparison of expectations between patients with shoulder complaints and patients with neck/back complaints. Several significant differences were found between the two groups in terms of patient characteristics. The patients with neck/back complaints had a statistically significantly higher level of emotional distress, as previously observed by Pincus et al. [37]. Patients with neck/back complaints also reported a lower education level and an increased NPS, the latter supporting the finding of Kalamari et al. [27]. An increased NPS could explain the elevated use of analgesics among the patients with neck/back complaints. When comparing expectations between the two patient groups, the patients with shoulder complaints appeared both to be more optimistic before the consultation and to a higher degree change their expectations in a positive direction during the consultation. Perhaps expectations are not subject to generalizations, and perhaps different patient groups should receive more individualized assessments of their expectations. However, expectations within various joint conditions are rarely compared in the literature.

The present study has several limitations that should be considered. Firstly, the included patients were primarily Norwegian, and invariance across cultures cannot be assumed. Considering the increased multicultural tendency in most Western countries, this limitation is perhaps unfortunate. Secondly, several PMR physicians conducted the consultations, and no adjustments were performed for their personalities, communication skills or clinical experience. Additionally, the physicians were not instructed in any particular way, and the different contents of the consultations were not taken into account. Perhaps recording of particular topics, such as addressing and/or discussion of expectations, could have provided insight into the detected changes in patient expectations. Thirdly, it is unfortunate that only sick leave, and not occupational status, was recorded. Fourthly, it is also a shortcoming that missing data in the non-completer group may have biased the comparison between completers and non-completers. It is uncertain why the non-completers had a shorter time of waiting between referral and the consultation. Fifthly, the general questionnaire, recording patient characteristics, was sent out along with the appointment letter and was filled out before the consultation, but the exact timing of this is uncertain, as the questionnaire was only returned on the appointment day. Finally, throughout the discussion, we have emphasized the unfortunate lack of comparable literature to challenge both the interpretation and the discussion of our results. The field of musculoskeletal expectations overall suffers from a shortage of tools for measuring expectations [38], which prevents systematization of the topic. Furthermore, “expectation” is a vague term that could be interpreted differently in different studies. We support Haanstra et al. [3] in calling for a more precise description of the term to optimize future research.

In our opinion, the current study is of note despite the mentioned limitations. Changes in expectations during a consultation have not been previously examined, and variations among patient groups have only been weakly addressed.

## Conclusion

This study suggests that expectations regarding pain and function change during or shortly after a PMR consultation. Approximately one quarter of patients attending their first PMR consultation change their expectations in a more positive direction after the consultation, while 10% of patients change their expectations in a negative direction. Hence, expectations can be influenced by a single specialist consultation. Among clinical prognostic factors, only sick leave was found to influence the changes in expectations. Overall, there is a lack of comparable literature, and more information will be required to more fully understand the potential clinical value of expectations.

## Additional files

Additional file 1: Questionnaire (Name: questionnaire.pdf, Title: Questionnaire. Description: questionnaire). (PDF 121 kb)
Additional file 2: Classification of diagnosis (Name: classification_diagnosis.pdf, Title: Classification of diagnosis, Description: Classification of neck, back and shoulder diagnosis). (PDF 123 kb)

## Abbreviations

GP: General practitioner
NRS: Numeric rating scale
OUS: Oslo University Hospital
PMR: Physical medicine and rehabilitation
PSOE: Patient Shoulder Outcome Expectancies
RCT: Randomized controlled trial

## References

[1] Rotter JB (1960). Some implications of a social learning theory for the prediction of goal directed behavior from testing procedures. Psychol Rev.

[2] Lurie JD, Berven SH, Gibson-Chambers J, Tosteson T, Tosteson A, Hu SS, Weinstein JN (2008). Patient preferences and expectations for care: determinants in patients with lumbar intervertebral disc herniation. Spine.

[3] Haanstra TM, Hanson L, Evans R, van Nes FA, De Vet HC, Cuijpers P, Ostelo RW (2013). How do low back pain patients conceptualize their expectations regarding treatment? Content analysis of interviews. Eur Spine J.

[4] Ozegovic D, Carroll LJ, David Cassidy J (2009). Does expecting mean achieving? The association between expecting to return to work and recovery in whiplash associated disorders: a population-based prospective cohort study. Eur Spine J.

[5] Janzen JA, Silvius J, Jacobs S, Slaughter S, Dalziel W, Drummond N (2006). What is a health expectation? Developing a pragmatic conceptual model from psychological theory. Health Expect.

[6] Kapoor S, Shaw WS, Pransky G, Patterson W (2006). Initial patient and clinician expectations of return to work after acute onset of work-related low back pain. J Occup Environ Med.

[7] Goldstein MS, Morgenstern H, Hurwitz EL, Yu F (2002). The impact of treatment confidence on pain and related disability among patients with low-back pain: results from the University of California, Los Angeles, low-back pain study. Spine J.

[8] O’Malley KJ, Roddey TS, Gartsman GM, Cook KF (2004). Outcome expectancies, functional outcomes, and expectancy fulfillment for patients with shoulder problems. Med Care.

[9] Foster NE, Bishop A, Thomas E, Main C, Horne R, Weinman J, Hay E (2008). Illness perceptions of low back pain patients in primary care: what are they, do they change and are they associated with outcome?. Pain.

[10] Bishop MD, Mintken PE, Bialosky JE, Cleland JA (2013). Patient expectations of benefit from interventions for neck pain and resulting influence on outcomes. J Orthop Sports Phys Ther.

[11] Mondloch MV, Cole DC, Frank JW (2001). Does how you do depend on how you think you’ll do? A systematic review of the evidence for a relation between patients’ recovery expectations and health outcomes. CMAJ.

[12] Iles RA, Davidson M, Taylor NF, O’Halloran P (2009). Systematic review of the ability of recovery expectations to predict outcomes in non-chronic non-specific low back pain. J Occup Rehabil.

[13] Myers SS, Phillips RS, Davis RB, Cherkin DC, Legedza A, Kaptchuk TJ, Hrbek A, Buring JE, Post D, Connelly MT (2008). Patient expectations as predictors of outcome in patients with acute low back pain. J Gen Intern Med.

[14] Goossens ME, Vlaeyen JW, Hidding A, Kole-Snijders A, Evers SM (2005). Treatment expectancy affects the outcome of cognitive-behavioral interventions in chronic pain. Clin J Pain.

[15] Mancuso CA, Graziano S, Briskie LM, Peterson MG, Pellicci PM, Salvati EA, Sculco TP (2008). Randomized trials to modify patients’ preoperative expectations of hip and knee arthroplasties. Clin Orthop Relat Res.

[16] Bialosky JE, Bishop MD, Cleland JA (2010). Individual expectation: an overlooked, but pertinent, factor in the treatment of individuals experiencing musculoskeletal pain. Phys Ther.

[17] Di Blasi Z, Harkness E, Ernst E, Georgiou A, Kleijnen J (2001). Influence of context effects on health outcomes: a systematic review. Lancet.

[18] Britten N, Stevenson FA, Barry CA, Barber N, Bradley CP (2000). Misunderstandings in prescribing decisions in general practice: qualitative study. BMJ.

[19] Cleary PD, McNeil BJ (1988). Patient satisfaction as an indicator of quality care. Inquiry.

[20] Hoffmann TC, Del Mar CB, Strong J, Mai J (2013). Patients’ expectations of acute low back pain management: implications for evidence uptake. BMC Fam Pract.

[21] Baker SC, Gallois C, Driedger SM, Santesso N (2011). Communication accommodation and managing musculoskeletal disorders: doctors’ and patients’ perspectives. Health Commun.

[22] Darlow B, Dowell A, Baxter GD, Mathieson F, Perry M, Dean S (2013). The enduring impact of what clinicians say to people with low back pain. Ann Fam Med.

[23] Skatteboe S, Roe C, Fagerland MW, Granan LP. Expectations for treatment outcomes in neck/back patients regarding improvements in pain and function: A cross-sectional pilot study. Eur J Phys Rehabil Med. 2014;50:649–56. 24755775

[24] Derogatis LR, Lipman RS, Rickels K, Uhlenhuth EH, Covi L (1974). The Hopkins Symptom Checklist (HSCL): a self-report symptom inventory. Behav Sci.

[25] Strand BH, Dalgard OS, Tambs K, Rognerud M (2003). Measuring the mental health status of the Norwegian population: a comparison of the instruments SCL-25, SCL-10, SCL-5 and MHI-5 (SF-36). Nord J Psychiatry.

[26] Nunnaly J (1978). Psychometric theory.

[27] Kamaleri Y, Natvig B, Ihlebaek CM, Benth JS, Bruusgaard D (2008). Number of pain sites is associated with demographic, lifestyle, and health-related factors in the general population. Eur J Pain.

[28] Stuart A (1955). A test for the homogeneity of the marginal distributions in a two-way classification.

[29] Green SB (1991). How many subjects does it take to do a regression analysis. Multivariate Behav Res.

[30] Boonstra AM, Reneman MF, Stewart RE, Schiphorst Preuper HR (2011). Do male and female patients with chronic musculoskeletal pain differ in their pre-treatment expectations of rehabilitation outcome?. J Rehabil Med.

[31] Hopayian K, Notley C (2014). A systematic review of low back pain and sciatica patients’ expectations and experiences of health care. Spine J.

[32] Georgy EE, Carr EC, Breen AC (2013). Back pain management in primary care: development and validity of the Patients’ and Doctors’ Expectations Questionnaire. Qual Prim Care.

[33] Sanderson KB, Roditi D, George SZ, Atchison JW, Banou E, Robinson ME (2012). Investigating patient expectations and treatment outcome in a chronic low back pain population. J Pain Res.

[34] Vasseljen O, Woodhouse A, Bjorngaard JH, Leivseth L (2013). Natural course of acute neck and low back pain in the general population: the HUNT study. Pain.

[35] Reme SE, Hagen EM, Eriksen HR (2009). Expectations, perceptions, and physiotherapy predict prolonged sick leave in subacute low back pain. BMC Musculoskelet Disord.

[36] Kongsted A, Vach W, Axo M, Bech RN, Hestbaek L (2014). Expectation of recovery from low back pain: a longitudinal cohort study investigating patient characteristics related to expectations and the association between expectations and 3-month outcome. Spine (Phila Pa 1976).

[37] Pincus T, Burton AK, Vogel S, Field AP (2002). A systematic review of psychological factors as predictors of chronicity/disability in prospective cohorts of low back pain. Spine (Phila Pa 1976).

[38] van Hartingsveld F, Ostelo RW, Cuijpers P, de Vos R, Riphagen II, de Vet HC (2010). Treatment-related and patient-related expectations of patients with musculoskeletal disorders: a systematic review of published measurement tools. Clin J Pain.

