# Supplementary material for: Expectations of pain and functioning in patients with musculoskeletal disorders: a cross-sectional study
Source: BMC Musculoskelet Disord. 2017 Jan 26;18:48. doi: 10.1186/s12891-016-1386-z (PMC5270237; doi:10.1186/s12891-016-1386-z)
Supplement: Additional file 1: — Questionnaire (Name: questionnaire.pdf, Title: Questionnaire. Description: questionnaire). (PDF 121 kb) [file 12891_2016_1386_MOESM1_ESM.pdf]

Oslo universitetssykehu

**YOUR EXPECTATIONS *(– before doctor appointment)***

(PSOE)

Compared with now, I think my **shoulder problem overall** next month will be:

□ Much worse □ Worse □ A little worse □ The same □ Better □ Much better

Compared with now, I expect my **shoulder pain** next month will be:

□ Much worse □ Worse □ A little worse □ The same □ Better □ Much better

Compared with now, I expect my **ability to use and move** my shoulder next month will be

□ Much worse □ Worse □ A little worse □ The same □ Better □ Much better

(11-NRS)

Describe your **neck and/or back problem(s)** as they are now:

**Pain at rest:**

No pain □ 1 □ 2 □ 3 □ 4 □ 5 □ 6 □ 7 □ 8 □ 9 □ 10 Worst pain imaginable

**Pain during activity** (all activities, including daily activities and exercise):

No pain □ 1 □ 2 □ 3 □ 4 □ 5 □ 6 □ 7 □ 8 □ 9 □ 10 Worst pain imaginable

Ability to **use as well as move your neck and/or back:**

No move- □ 1 □ 2 □ 3 □ 4 □ 5 □ 6 □ 7 □ 8 □ 9 □ 10 No move-ment restrictions
ment possible

**YOUR EXPECTATIONS - (– after doctor appointment)**

(PSFS.)

Compared with now, I think **my shoulder problem overall** next month will be:

□ Much worse □ Worse □ A little worse □ The same □ Better □ Much better

Compared with now, I expect my **shoulder pain** next month will be:

□ Much worse □ Worse □ A little worse □ The same □ Better □ Much better

Compared with now, I expect my **ability to use and move my shoulder** next month will be:

□ Much worse □ Worse □ A little worse □ The same □ Better □ Much better
