# Supplementary material for: Expectations of pain and functioning in patients with musculoskeletal disorders: a cross-sectional study
Source: BMC Musculoskelet Disord. 2017 Jan 26;18:48. doi: 10.1186/s12891-016-1386-z (PMC5270237; doi:10.1186/s12891-016-1386-z)
Supplement: Additional file 2: — Classification of diagnosis (Name: classification_diagnosis.pdf, Title: Classification of diagnosis, Description: Classification of neck, back and shoulder diagnosis). (PDF 123 kb) [file 12891_2016_1386_MOESM2_ESM.pdf]

Classification of neck/back diagnoses

ICD-10	**1. Specific**
	Neurogenic
	Other pathology (inflammation, cancer, trauma +)
40.0-.9	Deforming back conditions
41.0-.9	Scoliosis
42.0-.9	Spinal osteochondrosis
43.0-.9	Other deforming dorsopathies
45.0-.9	Ankylosing spondylitis
46.0-.9	Inflammatory conditions
47.0-9	Spondylosis
48.0-.9	Other spondylopathies
49.0-.9	Spondylopathies in diseases classified elsewhere
50.0-.9	Cervical disk disorder
51.0-.9	Other intervertebral disc disorder
53.9	Dorsopathy, unspecified
54.0	Panniculitis affecting regions of neck and back
XIX	Injury, poisoning and other consequences of external causes
	**2. Unspecific**
	Unspecific back-pain
48.8	Other specified spondylopathies
50.9	Cervical disc disorder, unspecified
51.9	Intervertebral disc disorder, unspecified
53.0	Cervicocranial syndrome
53.1	Cervicobrachial syndrome
53.2	Spinal instabilities
53.3	Sacrococcygeal disorders, not elsewhere classified
53.8	Other specified dorsopathies
54.1	Radiculopathy
54.2	Cervicalgia
54.3	Sciatica
54.4	Lumbago with sciatica
54.5	Low back pain
54.6	Pain in thoracic spine
54.8	Other dorsalgia
54.9	Dorsalgia, unspecified
25.5	Pain in joint
79.1	Myalgia

Comments: Specific - unspecific neck/back diagnoses:
- There is a code .9 in many chapters, which are considered specific. These diagnoses have a defined origin, since they are ordered in a chapter-
- "Diseases classified elsewhere" are considered the same way.
- In cases of doubt where the patient journal evaluated.

Source: www.formi.no

Classification of shoulder diagnoses

ICD-10	Group 1
	**SUBACROMIAL CONDITIONS**
75	*Shoulder lesions*
75.1	Rotator cuff syndrome
75.2	Bicipital tendinitis
75.3	Calcific tendinitis of shoulder
75.4	Impingement syndrome of shoulder
75.5	Bursitis of shoulder
	Group 2
	**ADHESIV CAPSULITIS**
75.0	Adhesive capsulitis of shoulder
	Group 3
	**DEGENERATIVITY/ARTROSIS**
15-19	Arthrosis
15.0-.9	Polyarthrosis
19.0-.9	Other arthrosis
	Group 4
	**OTHER CONDITIONS**
0-3	***Infectious arthropathies***
00.0-.9	Pyogenic arthritis
01.0-.9	Direct inf. of joint in inf./parasitic dis. clas. els.
02.0-.9	Reactive arthropathies
03.0-.9	Postinfective and reactive
0.5-14	***Inflammatory polyarthropathies***
0.5-.9	Seropositive rheumatoid arthritis
0.6-.9	Other rheumatoid arthritis
0.7-.9	Psoriatic and enteropathicarthropathies
0.8-.9	Juvenile rheumatoid arthritis
10.0-.9	Gout
11.0-.9	Other crystal arthropathies
12.0-.9	Other specific arthropathies
13.0-.9	Other arthritis
14.0-.9	Arthropathies in other diseases clas. els.
20-25	***Other joint disorders***
30-36	***Systemic connective tissue disorders***
30.0-.9	Polyarteritis nodosa
31.0-.9	Other necrotizing vasculopathies
32.0-.9	Systemic lupus erythematosus

33.0-.9	Dermatopolymyositis
34.0-.9	Systemic sclerosis
35.0-.9	Other systemic involvement of connective tissue
36.0-.9	Systemic connective tissue in dis. clas. els.
60-79	***Soft tissue disorders***
60-63	***Disorders of muscles***
60.0-.9	Myositis
61.0-.9	Calcification and ossification of muscle
62.0-.9	Other disorders of muscle
63.0-.9	Disorders of muscle in diseases clas. els.
65-68	***Disorders of synovium and tendon***
65.0-.9	Synovitis and tenosynovitis
66.0-.9	Spontaneous rupture of synovium and tendon
67.0-.9	Other disorders of synovium and tendon
70-79	***Other soft tissue disorders***
70.0-.9	Soft tissue disorders related to use, overuse
71.0-.9	Other bursopathies
72.0-.9	Fibroblastic disorders
73.0-.9	Soft tissue disorders indiseases clas. els.
75.8	Other shoulder lesions
75.9	Shoulder lesion, unspecified
79.0	Other soft tissue disorders, not els. clas.
80-84	***Osteopathies***
80.0-83.9	Osteoporosis with pathological fracture
84.0	Malunion of fracture
85.0	Fibrous dysplasia
86-90	*Other boneconditions*
86.0-.9	Osteomyelitis
87.0-.9	Osteonecrosis
88.0-.9	Paget's disease of bone
89.0-.9	Algoneurodystrophy
90.0-.9	Osteopathies in diseases clas. els.
91-94	***Chondropathies***
93.0-94.9	Other osteochondropathies
95-99	***Other disorders of the MSS and connective tissue***
95.0-.9	Acquired deformities
96.0-.9	Pseudarthrosis after fusion or arthrodesis
99.0-.9	Biomechanical lesions, not clas. els.
XIX	Injury, poisoning and other consequences of external causes
S40-49	Injuries to the shoulder and upper arm

clas. els.: classified elsewhere

Source:

- Niels Gunnar Juel
- Cecilie Røe
